# Correction: A reproducible approach for the use of aptamer libraries for the identification of Aptamarkers for brain amyloid deposition based on plasma analysis

**DOI:** 10.1371/journal.pone.0320967

**Published:** 2025-04-30

**Authors:** Cathal Meehan, Soizic Lecocq, Gregory Penner

In Fig 1, the random nucleotides in the template should have been highlighted in green. Please see the correct Fig 1 here,

**Fig 1:**
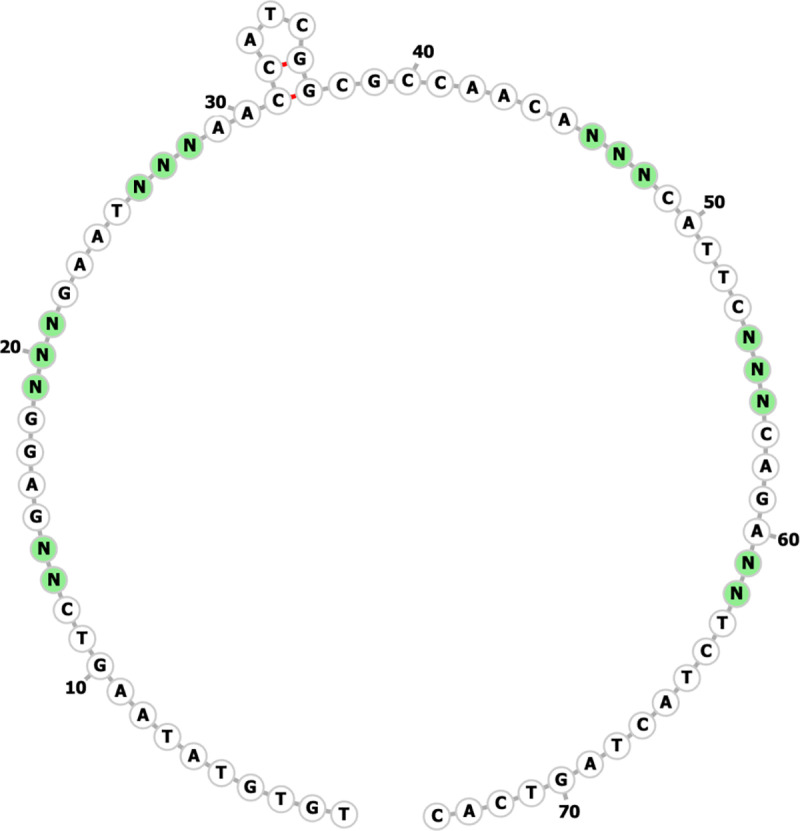
Predicted secondary structure of fixed sequences in Neomer library.

## References

[1] Meehan C, Lecocq S, Penner G. A reproducible approach for the use of aptamer libraries for the identification of Aptamarkers for brain amyloid deposition based on plasma analysis. PLoS One. 2024;19(8):e0307678. doi: 10.1371/journal.pone.0307678 39190656 PMC11349097

