# Supplementary figures and images for: A reproducible approach for the use of aptamer libraries for the identification of Aptamarkers for brain amyloid deposition based on plasma analysis
Source: PLoS One. 2024 Aug 27;19(8):e0307678. doi: 10.1371/journal.pone.0307678 (PMC11349097; doi:10.1371/journal.pone.0307678)

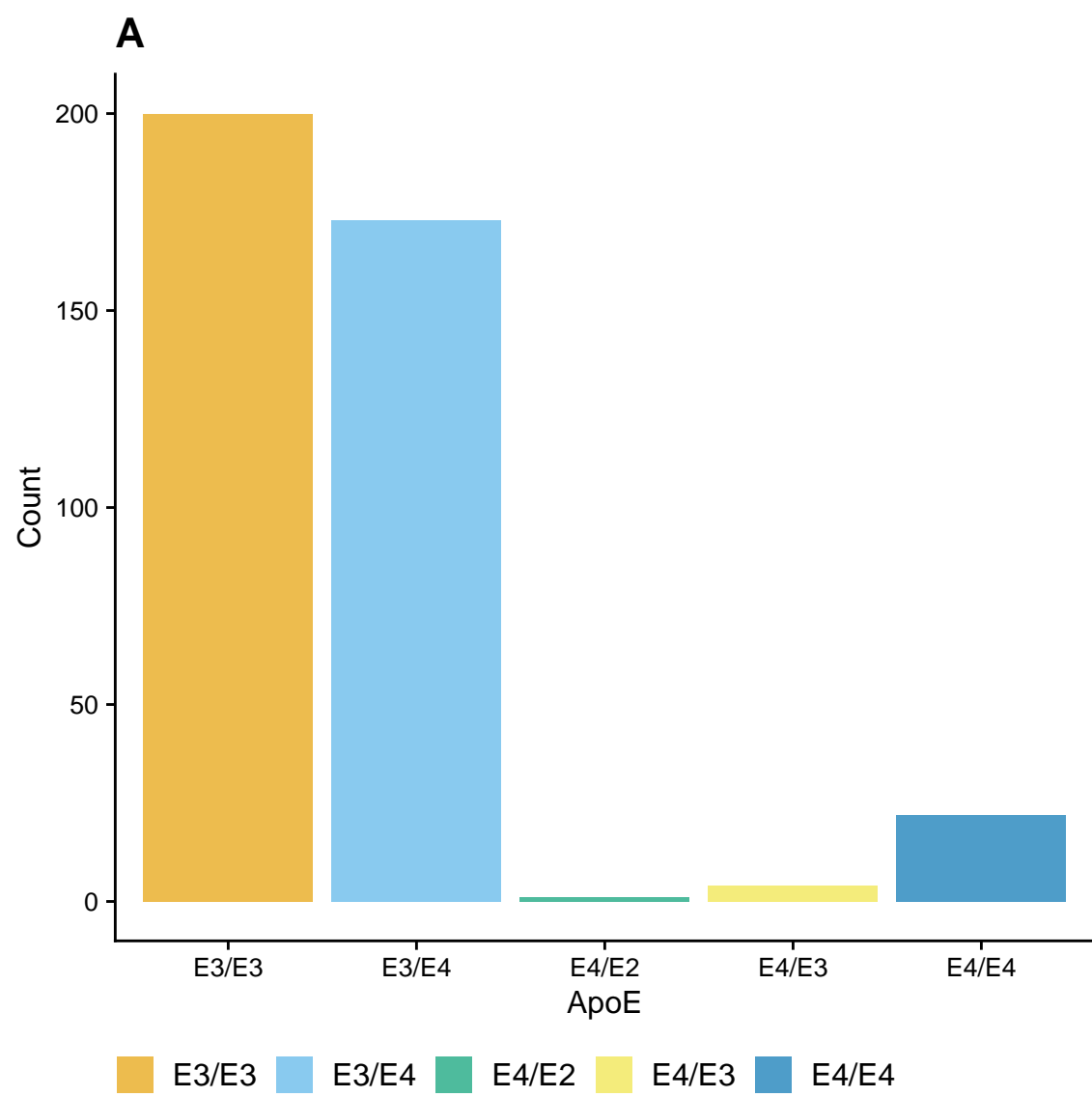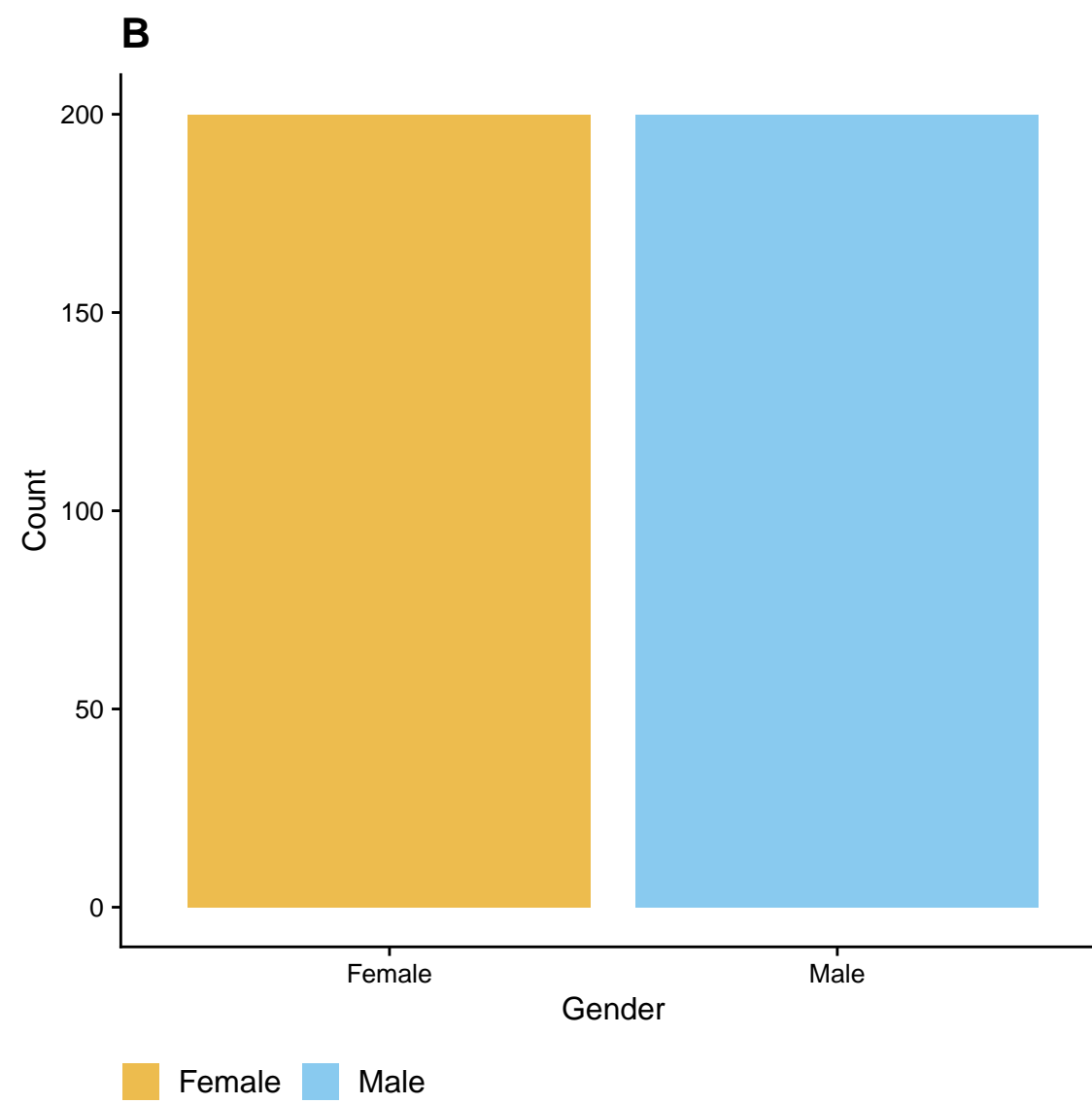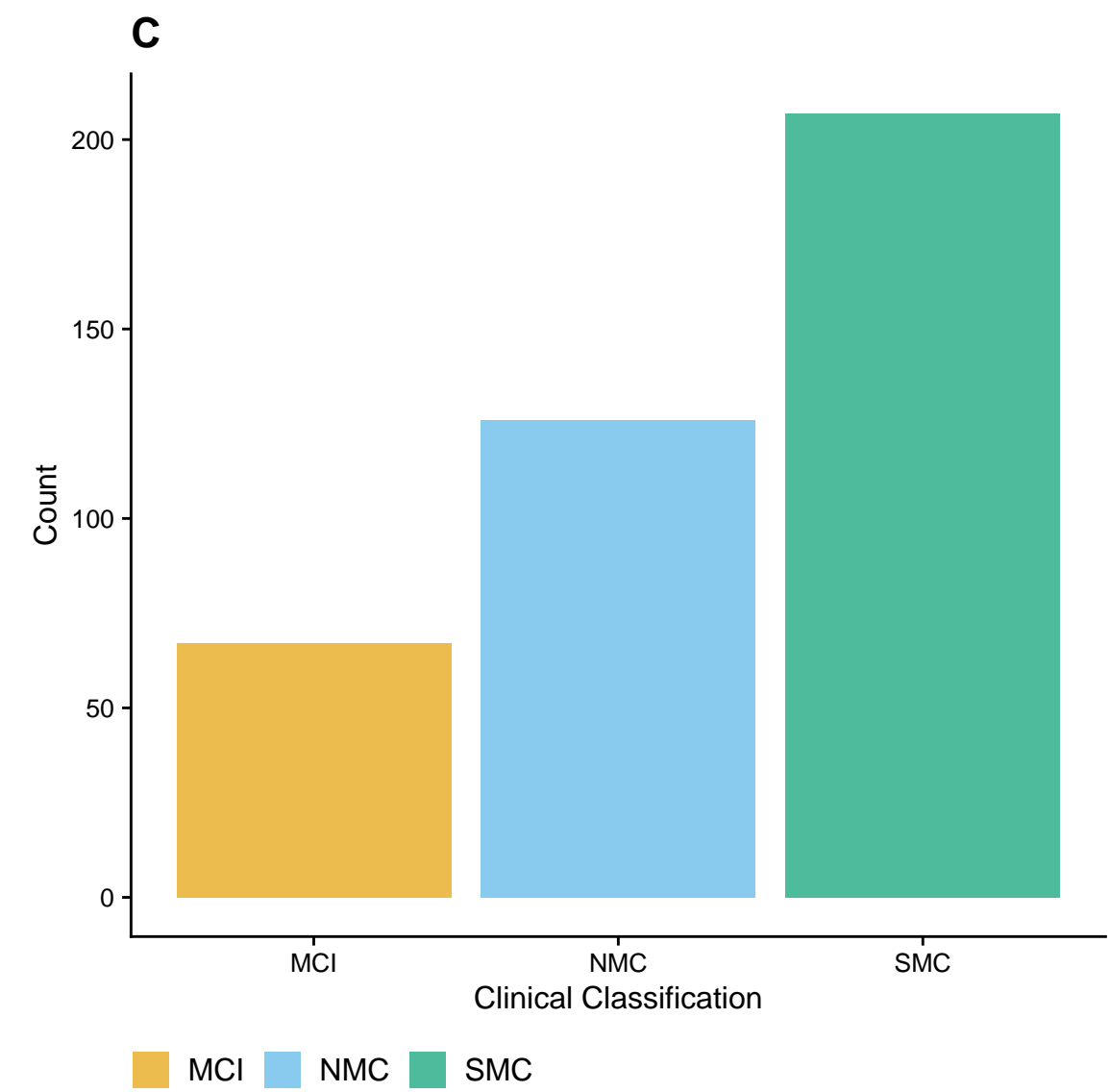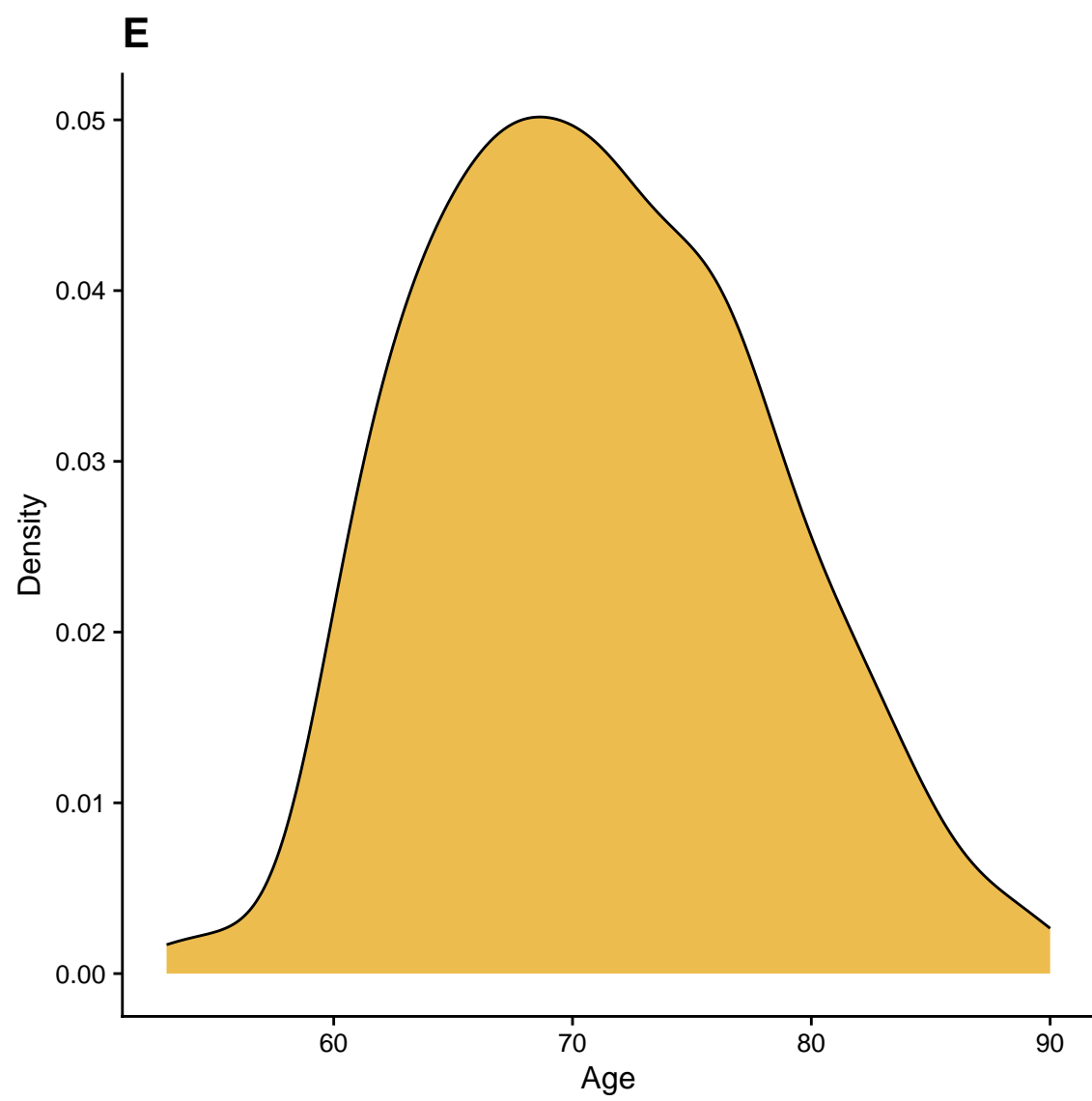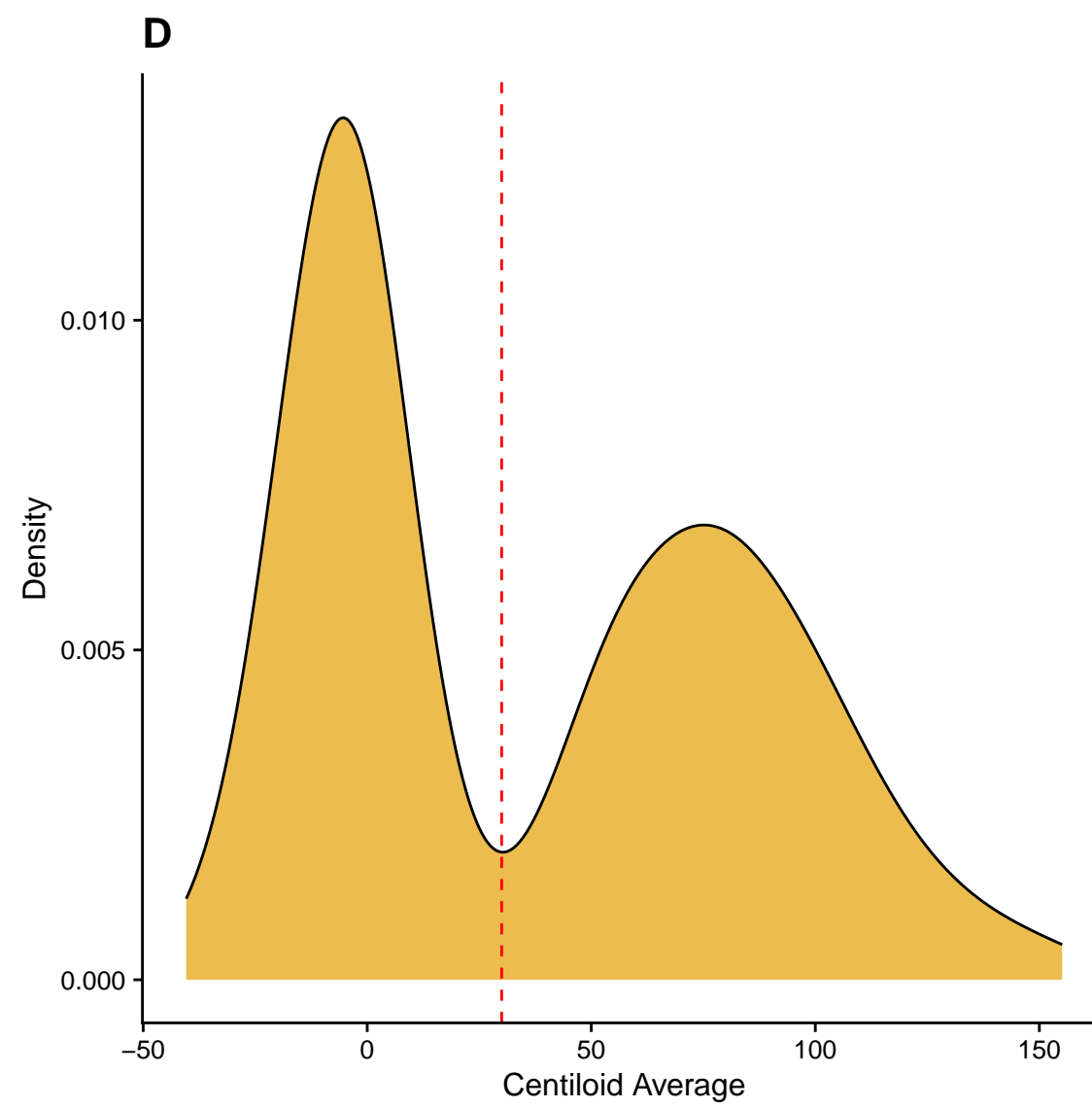

Supplement: S1 Fig — (PDF) [file pone.0307678.s001.pdf]
