# Supplementary material for: A reproducible approach for the use of aptamer libraries for the identification of Aptamarkers for brain amyloid deposition based on plasma analysis
Source: PLoS One. 2024 Aug 27;19(8):e0307678. doi: 10.1371/journal.pone.0307678 (PMC11349097; doi:10.1371/journal.pone.0307678)
Supplement: Attachment — Submitted filename: Response to reviewers.docx [file pone.0307678.s005.docx]

**Response to Reviewer #1:**

The reviewer has misunderstood the methodology employed in this study for identifying aptamers and developing predictive models. To clarify, the Neomer library and data analysis method were utilized to identify aptamers binding to unknown biomarkers enriched in individuals with high and low amyloid levels. Subsequent qPCR assays on plasma samples using these aptamers facilitated the development of machine learning models with enhanced predictive capacity compared to traditional SELEX-selected aptamers. This distinction is explicitly stated in the manuscript.

However, to avoid confusion between techniques, the SELEX comparison has been omitted.Regarding statistical significance, the 20% increase in sensitivity observed with 99 samples is deemed an improvement over previous methods while maintaining specificity. Nonetheless, the SELEX comparison has been removed to mitigate confusion. The manuscript has been edited to enhance readability and maintain clarity of objectives.

**Response to Reviewer #2:**

Real world applications seem to be a large concern for reviewer 2. To address these concerns, a pre-print demonstrating real-world application scenarios has been submitted to Medrxiv that we plan to submit for publication after this paper. This manuscript focuses on introducing the concept of using aptamarkers to identify predictive biomarkers for Alzheimer’s disease. We could reference and discuss this in the text but we would prefer to respect the chronology of the research and development carried out by the lab. Minor issues of figures and tables have been addressed by removing the SELEX comparison.

https://www.medrxiv.org/content/10.1101/2023.11.15.23298582v1.article-info

**Response to Reviewer #3:**

The manuscript has been rewritten for clarity by removing the SELEX comparison. Despite this, it remains puzzling how the reviewers find sensitivity, specificity, and AUROC insufficient to evaluate the predictive efficacy of machine learning models using aptamarkers. These metrics are industry-standards in assessing the accuracy and reliability of applications in diagnostics.
