# Supplementary material for: A reproducible approach for the use of aptamer libraries for the identification of Aptamarkers for brain amyloid deposition based on plasma analysis
Source: PLoS One. 2024 Aug 27;19(8):e0307678. doi: 10.1371/journal.pone.0307678 (PMC11349097; doi:10.1371/journal.pone.0307678)
Supplement: Attachment — Submitted filename: Response to reviewers 19.6.24.docx [file pone.0307678.s006.docx]

Dear Editor,

Thank you for considering our manuscript "A reproducible approach for the use of aptamer libraries for the identification of Aptamarkers for brain amyloid deposition based on plasma analysis" for publication in PLOS One. We appreciate the constructive feedback from the reviewers. We have revised the manuscript to address their comments as detailed below. The changes have also been made to comply with PLOS One's formatting guidelines.

Reviewer #1:

1. The quality of the figures are poor, I suggest revising the figures in 600dpi for clear visibility. Response: All figures have been improved to higher resolution as suggested.
2. The conclusion section is insufficient to summarize the proposed study, I suggest revising. Response: The conclusion has been extensively expanded to better summarize the study and its implications.
3. The hyper-parameters used for training the proposed model should be provided in the form of a table. Response: A table of model hyperparameters has been added to the supplementary materials.
4. I suggest providing a discussion section to demonstrate the effectiveness of the achieved results and its impacts. Response: Additional discussion has been added regarding the significance and impact of the results.
5. The feature selection section is not clear.
   Response: The feature selection methodology has been clarified on page 17. Default hyperparameters were used and less predictive features were sequentially excluded. More extensive explanations have been added.
6. For user concerns, I suggest adding the recent predictors such as DeepAVP-TPPred,Deepstacked-AVPs, iAFPs-Mv-BiTCN,pAtbp-EnC, and pAVP_PSSMDWT-EnC. Response: These recent predictors are now discussed and referenced in the manuscript.
7. At the introduction section I suggest adding the contributions of the proposed study in points. Response: The contributions are now discussed in greater detail in the introduction and conclusion, though not in point form.
8. What should be future directions of the proposed study.
   Response: A future directions section has been added.

Reviewer #2:

1. The plasma samples were collected from twenty individuals without any selection criteria (including exclusion and inclusion) of people, that participated in the study. I did not find any ethical committee statement indicating approval of this research.
   Response: We have clarified that the samples came from the well-characterized AIBL cohort. The diagnoses, selection criteria and ethical approvals for this cohort are now referenced in the introduction when the AIBL cohort is first mentioned.
2. There are differences in the Abstract and Results section regarding the clinical variables used in the model. Response: This inconsistency has been corrected. Three clinical variables (age, sex, clinical status) were used as stated in the Results.
3. Please, provide the set of descriptors as supplementary materials, for instance in CSV, TXT or ARFF files. It would be great to provide the graphical representation of the descriptors as they are used in the study.
   Response: The descriptor set and plots have been added to the supplementary materials as suggested.
4. What does "Train accuracy" mean in Table 3? Would it be possible to include also sensitivity and specificity for the training set?
   Response: The table heading has been changed to "Training accuracy". Sensitivity and specificity for the training set have been added to the supplementary materials.
5. Figures and Tables formatting should be carefully checked and corrected. Response: All figures and tables have been reformatted to comply with PLOS One guidelines, as detailed below.

Reviewer #3:

1. I think a descriptive table of the sample in terms of age, sex amyloid and clinical status should be provided. Response: This descriptive table has been added to the supplementary materials.
2. According to the authors, "Table 3 provides a summary of all algorithms that returned perfect predictive results on the test set." Do they mean training set? It seems to me that no model achieved perfect predictions in the test set.
   Response: This has been corrected to refer to the training set performance.
3. In general, in the results section, I'm not always clear what model they are referring to in what sentence, as they generally seem to refer to the best performer but sometimes they comment on other models as well. Maybe just assign a number or a letter to each model to refer to them univocally in text. Also in figure 6 I assume the ROC refers once again to the best performer, still the numbers don't match: AUROC 0.78 vs 0.79. This may be due rounding error, in which case it needs to be fixed. Response: Reference numbers have been added for each ML model to improve clarity. The AUROC discrepancy in Figure 6 has been fixed.
4. The authors report sensitivity, specificity and accuracy, but they don't mention how the cutoff value used to calculate those metrics was chosen. Response: The probability cutoff selection method has been specified in the Materials and Methods under Model Evaluation.
5. I would like to see confidence intervals for all performance metrics.
   Response: 95% confidence intervals for the performance metrics have been added to the supplementary materials.
6. Figure 5 is a bit too vague to me, it doesn't even have a y-axis label. Please specify what metrics are uses for feature importance. Response: Figure 5 has been updated with a more detailed legend and y-axis label specifying the feature importance metric.

Formatting changes: The manuscript has undergone extensive reformatting to comply with PLOS One guidelines, as detailed in the accompanying note from Kiera Drew. Key changes include:

- References reformatted to PLOS One style, using "et al" for sources with 6+ authors
- Figure titles shortened to <15 words and placed in bold above figures
- Table titles moved above tables
- Supplementary figure and table names and titles modified
- Equation for qPCR efficiency formatted with Word's equation tool
- Acknowledgements section added
- Abstract simplified to contain only one abbreviation (AUC)
- Short title of 50 characters to be entered during submission process

Please see the marked-up and clean versions of the revised manuscript, as well as the PLOS One formatting guide, for full details of the changes made.

Additionally we’ve addressed other comments on the Funding and Competiting Interests statements, funding information and data availability as clear as we possibly can. We can’t find a section for adding this on the online submission of the revision, so we’ve included this as a word documents in the attached files.

We hope that these revisions and reformatting address the reviewers' comments and meet PLOS One's publication standards. Thank you again for your consideration.
